# Social media interventions for autistic individuals: Systematic review

**DOI:** 10.3389/fpsyt.2023.1089452

**Published:** 2023-03-01

**Authors:** Elia Gabarron, Ingjerd Skafle, Anders Nordahl-Hansen, Rolf Wynn

**Affiliations:** ^1^Department of Education, ICT and Learning, Østfold University College, Halden, Norway; ^2^Norwegian Centre for E-health Research, University Hospital of North Norway, Tromsø, Norway; ^3^Faculty of Health, Welfare and Organisation, Østfold University College, Fredrikstad, Norway; ^4^Faculty of Medicine, University of Oslo, Oslo, Norway; ^5^Department of Clinical Medicine, UiT The Arctic University of Norway, Tromsø, Norway

**Keywords:** social media, intervention studies, autism (ASD), interventions, autistic, disability

## Abstract

**Background:**

Research on the use of digital technologies for delivering behavioral interventions has shown mixed evidence on their efficacy for improving both autistic symptoms and co-occurring psychiatric disorders. Little knowledge exists on the specific use or efficacy of using social media in interventions aimed at autistic individuals.

**Objective:**

To review and describe the current existing evidence-based research on the use of social media in interventions aimed at autistic individuals.

**Methods:**

A search was conducted across 8 databases (PubMed; EMBASE; Cochrane Library; PsycInfo; ERIC; Education Source; Web of Science; and IEEE Xplore). We included primary studies and reviews that dealt with autism spectrum disorder (ASD); described interventions that use social media; and reported results from the intervention. The quality of the evidence of the included primary studies was graded according to the GRADE criteria, and the risk of bias in systematic reviews was assessed by drawing on the AMSTAR guidelines. Results were synthesized and sorted by quality of evidence.

**Results:**

A total of nine articles were included in this review: eight primary studies (five non-randomized interventions and three randomized interventions) and one systematic review. The total number of participants with an ASD-diagnosis in the included studies was 164 (aged 5 to 22 years old). Studies weighted as being of moderate quality of evidence have reported significant positive effects in the groups that received the social media interventions: increased social engagement and participation in life situations; increased physical activity level; increased improvement on occupational performance, specified goals, and behavioral problems; and decreased plaque scores coupled with parent reports of intervention success. None of the studies have reported any negative effects linked to social media interventions.

**Conclusion:**

There is very little evidence of good quality on the use of social media in interventions aimed at autistic individuals. While there is a need for more high-quality studies, all the included studies, with one exception found positive results of the interventions. These findings are encouraging, suggesting that social media-based interventions may in fact be useful for supporting behavioral changes in autistic individuals.

**Systematic review registration:**

https://www.crd.york.ac.uk/PROSPERO/display_record.php?RecordID=337185, identifier CRD42022337185.

## Introduction

Individuals diagnosed with autism Spectrum Disorder (ASD) have persistent deficits in areas of social communication and interaction, plus restricted and repetitive behaviors (1). Diverse types of interventions are described in the literature addressing these core-defining features of ASD. Evidence-based research shows that behavioral interventions are beneficial for improving some of the characteristics of autism, such as cognitive ability (2, 3), and motor skills (2) in autistic children. And while some research has found that these interventions have not proven their efficacy for improving communication (2–5), adaptative behavior (2, 4, 5), socialization (2, 4, 5), or autism general symptoms (4); other studies have found significant improvements linked to early interventions on expressive language (5, 6), and daily living skills (2, 3). Exercise interventions have also proved to be beneficial for reducing unwanted stereotyped motor behavior in children (7).

As with traditional interventions, recent research on the use of digital technologies for delivering behavioral interventions has shown mixed evidence on their efficacy for improving both autistic symptoms and co-occurring psychiatric disorders. Evidence exists on the benefit of using computers, tablets, apps, or other information and communication technologies for improving social skills (8–10), social behavior (11), social communication (11), or facial emotion recognition (12) in individuals with ASD. But evidence also shows that some of these digital technologies do not have an effect on improving social communication skills in children with ASD (13). The research on the use and importance of social media has increased significantly in recent years. Social media might be used in interventions addressed to autistic individuals. Social media interventions refer to the use of social media channels or functionalities in any type of intervention. Social media interventions have proven their efficacy for improving several outcomes in a number of other conditions (14–20). The use of social media interventions for this group has a high potential as both autistic adolescents and youth (21–24), as well as autistic adults are using these media in their daily lives (25, 26). There is a wide debate around autism on social media (27, 28), and a big community of autistic individuals uses these channels (29, 30). However, little knowledge exists on the use or efficacy of using these media in interventions aimed at individuals with ASD.

The objective of this paper is to review and describe the current evidence-based research on the use of social media in interventions addressed to autistic individuals.

## Materials and methods

We have performed a systematic review to capture the current evidence on the use of social media in interventions related to autistic individuals. We had two research questions: (1) Is there evidence on the use of social media in interventions aiming at autistic children and adults?; and (2) What are the reported outcomes (health; mental health; behavioral; educational; other outcomes) of these social media interventions in comparison with usual practice?

This review followed the Preferred Reporting Items for Systematic Reviews and Meta-Analyses (PRISMA 2020 Statement) (31) and the Measurement Tool to Assess systematic Reviews (AMSTAR) guidelines (32).

### Search strategy and information sources

To answer the research questions, an electronic search was carried out on July 4th, 2022. This first search was carried out by the first author. The search covered published studies comprising the **terms related to social media** “Social media” OR “Social networking” OR any of the top 20 world’s most popular social networks (33) “Facebook” OR “YouTube” OR “WhatsApp” OR “Messenger” OR “Instagram” OR “WeChat” OR “Kuaishou” OR “TikTok” OR “Telegram” OR “Qzone” OR “QQ” OR “Weibo” OR “Douyin” OR “Snapchat” OR “Twitter” OR “Pinterest” OR “Reddit” OR “LinkedIn” OR “Quora” OR “Skype” in combination with **terms related to autism** (“Autism Spectrum Disorder” OR “Autistic Disorder” OR “Autism” OR “Autistic” OR “ASD” OR “Asperger Syndrome” OR “Asperger” OR “Pervasive developmental disorder” OR “Pervasive development disorder” OR “PDD” OR “PDD-NOS” OR “Neurodevelopmental disorder”) included **in the title or abstract** and indexed in the following eight databases: PubMed, EMBASE, Cochrane Library, PyscInfo, ERIC, Education Source, Web of Science, and IEEE Xplore. **No year or language limitations** were used.

To detect possible new publications, a librarian specialist repeated the searches 3 months after the first search (4th October 2022). When possible this second search focused on articles published on or after the 5th of July 2022. The full search strategy is summarized in Supplementary Appendix 1.

### Eligibility criteria and selection process

Publications were included in the review if they fulfilled four criteria: (a) dealt with ASD (i.e., autistic individuals were specifically mentioned as target group); (b) described interventions that use social media; and (c) reported results from the interventions. Both primary studies and reviews were considered of interest and were therefore included in this review. Publications that did not meet all inclusion criteria were excluded from the review.

All references captured by the search engine were uploaded to EndNote 20 and Rayyan. Duplicates were identified and removed. To assess the eligibility of the papers two passes were done. In the first pass, all titles and abstracts were examined by two independent reviewers (EG and IS). On a second pass, the full text of the selected articles was extracted and carefully analyzed to confirm their eligibility by two independent reviewers (EG and AN-H). Discrepancies were resolved by a third reviewer (RW). The selected articles were included in the quality assessment.

### Data items and data extraction

The following data were extracted and analyzed: study design; interventions (focus, duration, and participants); used social media; intervention components according to the Behavior Change Wheel (BCW) framework (34); and effects of the interventions. Additionally we identified in each of the included primary studies the reporting of the 17 essential recommended by CONSORT-EHEALTH standards on reporting social media interventions (items 1ai “Identify the mode of delivery in the title”; 1aiii “Mention primary condition or target group in the title”; 1b “Mention key features/functionalities/components of the intervention and comparator in the abstract”; 2ai “Describe the problem and the type of system/solution that is object of the study”; 2aii “Describe what is known about the (type of) system that is the object of the study”; 4aii “Mention how participants were recruited”; 4bi “Clearly report if outcomes were (self-)assessed through online questionnaires”; 5vii “Describe how participants accessed the application”; 5viii “Describe mode of delivery, features/functionalities/components of the intervention and comparator, and the theoretical framework”; 5xi “Report any prompts/reminders used”; 5xii “Describe any co-interventions”; 11ai “Specify who was blinded, and who was not”; 12ai “Specify imputation techniques to deal with attrition/missing values”; 15i “Report demographics associated with digital divide issues”; 16i “Report multiple ‘denominators’ and provide definitions”; 20i “Discuss Typical limitations in ehealth trials”; and 22i “Restate study questions and summarize the answers suggested by the data”) (35).

A single reviewer (EG) extracted the data from the included articles; and a second reviewer (AN-H) verified the appropriateness of the extracted data.

### Coding

Intervention components of primary studies were coded according to the BCW framework (34). The BCW framework was chosen because it uses an overarching model of behavior that integrates 19 frameworks for classifying behavior change interventions into a single one, and provides a clear structure for categorizing intervention functions (34). The BCW framework identifies 9 intervention components that can be implemented for changing behavior (34). Intervention components were coded according to this framework into: Education (intervention uses components to increase knowledge); Persuasion (intervention uses communication to induce stimulate action); Incentivization (intervention incorporates expectations of reward); Coercion (intervention incorporates expectations of punishment); Training (intervention impart skills); Restriction (intervention uses techniques to reduce the opportunity of engaging in the target behavior); Environmental restructuring (intervention includes changes in physical environment); Modeling (intervention provides examples for people to imitate); and Enablement (intervention increases means or reduce barriers to increase capability) (34).

Interventions components were coded by a single reviewer with a background in psychology (EG) and verified by a second reviewer with a background in psychiatry (RW).

### Quality evidence assessment and risk of bias

The quality of evidence and risk of bias of the studies included in this review were classified by two independent reviewers (EG and RW). The quality of evidence of primary studies was assessed following the GRADE guidelines (36). The risk of systematic bias was assessed by drawing on the AMSTAR criteria (32). Only primary studies and reviews that included primary studies were included in the qualitative synthesis.

## Results

### Study selection

**FIGURE 1:**
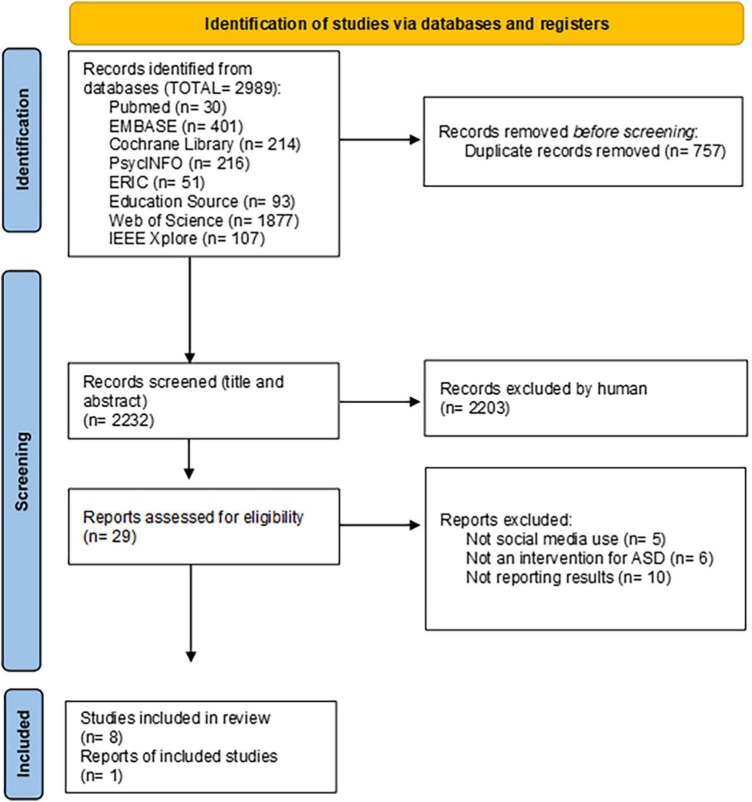
Flowchart of the selection procedure.

A total of 2,989 records were identified in both data searches. After removing duplicates, 2,232 titles and abstracts were screened, and of those, eight articles met the inclusion criteria (37–44). We searched the reports of trial registrations and one additional report was thereby identified and added (45). Therefore, the final number of included studies in this review was 9 (see Figure 1). The list of relevant studies that were read in full text but were excluded from the review and the reasons for exclusion can be found in Supplementary Appendix 2.

### Description of the included studies

The main characteristics of the nine included studies are presented in Table 1.

**TABLE 1:** Summary of the studies included in this review (*n* = 9).

References	Study design	Focus of the intervention (primary outcomes measures)	Intervention duration	Study participants	Social media	Used BCW intervention components* Michie et al. (34)	Findings	Quality of evidence
Saxena et al. (43)	Systematic review	Provide information and social support (effectiveness of mentorship programmes -several measures-)	Several, from 6 h to 79 months	*n* = 721 children/adolescents with ASD and other neurodevelopmental disabilities (aged 10–19)	WhatsApp, Facebook	N/A	Online peer mentorship programmes have positive influence on social engagement and participation in life situations for children and adolescents with disabilities (Cohen’s *d* = 0.55–1.74).	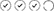 Moderate^a^
Popple et al. (45)	Randomized intervention	Education on how to brush teeth (oral hygiene and plaque index)	3 weeks	*n* = 18 children/adolescent with ASD (aged 5–14)	YouTube	1, 5, 8	Teeth hygiene marginally improved in both groups (non-significant). Decreased plaque scores in the experimental group [MC = 1.2 (1.05), ME = 0.38 (0.43); *d* = 1.02], coupled with parent reports of intervention success	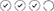 Moderate^b^
Yarimkaya et al. (44)	Randomized intervention	Increase physical activity (physical activity level)	6 weeks	*n* = 42 families and their child with ASD (66.7% boys. Mean age: 5.5–5.9 years)	WhatsApp, YouTube	1, 2, 3, 5, 7, 8, 9	Significant increase in the physical activity level in the experimental group ANOVA: [*F*_(1,40)_ 1/4 37.843; *p* < 0.05]. Families satisfied with the intervention (parents reported increasing levels of physical activity; promoting family participation; improving movement skills; and reducing technological tool addiction)	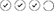 Moderate^b^
Jamali et al. (42)	Randomized intervention	Improve occupational performance (Canadian occupational performance measure)	16 sessions (2 sessions per week)	*n* = 43 children with ASD and their families (76.7% boys, Mean age: 8.33 years)	WhatsApp	1, 5	Significant greater improvement on occupational performance (COPM-performance partial η2 = 0.21; COPM-satisfaction partial η2 = 0.24), specified goals, and behavioral problems in the intervention group	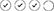 Moderate^b^
Esenturk and Yarimkaya (38)	Non-randomized intervention	Increase physical activity (feasibility questionnaire)	4 weeks	*n* = 14 parents and their child/adolescent with ASD (57.1% boys; Mean age: 12.07 years)	WhatsApp	1, 2, 3, 5, 7, 8, 9	Parents reported that WhatsApp-based physical activities were a feasible intervention to increase the physical activity level of their children with ASD and stated that the contents of the physical activity shared in the WhatsApp group were useful (no effect sizes were reported).	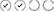 Low-very low^b^
Gwynette et al. (39)	Non-randomized intervention	Social skills (social responsiveness scale-2; and social skills improvement system rating scale)	8 weeks	*n* = 6 adolescents with ASD, all boys with normal IQ (aged 12–19)	Secret Facebook group	1, 5	No differences in social responsiveness or social skills (no effect sizes were reported). The Facebook intervention was well received by participants and their parents.	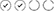 Low-very low^b^
Healy et al. (41)	Non-randomized intervention	Increase physical activity (perception of physical activity intervention)	4 weeks	*n* = 13 families with a child/adolescent with ASD (aged 6–16)	Facebook private group	1, 3, 8	Parents reported an overall positive perspective of the intervention as a scalable, sustainable, and economical means of intervention; and as a source of motivation, a reminder for them to take action and as a source of social support (no effect sizes were reported).	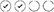 Low-very low^b^
Healy and Marchand (40)	Non-randomized intervention	Increase physical activity (feasibility measures)	4 weeks	*n* = 13 families with a child/adolescent with ASD (aged 6–16)	Facebook private group	1, 3, 8	All parents reported that they were satisfied or very satisfied with their overall experience of the project (no effect sizes were reported).	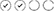 Low-very low^b^
Agganis (37)	Non-randomized intervention	Safety and social skills on social media (quality of the reply and report to lures)	315 min training package	*n* = 3 high school social media users with ASD, all male (aged 19–22)	Facebook	1, 5	Increased social media safety skills in all participants (Tau-*U*: Aggregate Effect Size: 0.7010). Skills maintained after training package was removed, at 7, 14, and 21 days	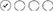 Very low^b^

*BCW intervention components Michie et al. (34): 1-education; 2-persuasion; 3-incentivization; 4-coercion; 5-training; 6-restriction; 7-environmental restructuring; 8-modeling; and 9-enablement. ^a^Quality of evidence and risk of bias assessment drawing on AMSTAR 2 criteria Shea et al. (32). ^b^Quality of evidence according to GRADE guidelines Guyatt et al. (36).

Among the nine included articles, five were non-randomized interventions (37–41), three were randomized interventions (42, 44, 45), and one was a systematic review (43).

Five studies were carried out in the US (37, 39–41, 45); two in Türkiye (38, 44), one in Iran (42), and the systematic review was carried out by authors from Canada, Poland, and Switzerland (43).

The eight included primary studies reported between 9 and 16 of the 17 essential items recommended by CONSORT-EHEALTH on social media interventions (35). Essential item #12 “Imputation techniques to deal with attrition or missing values” of CONSORT-EHEALTH was not reported by any of the included primary studies. The least reported items were both item #5 “Report any prompts/reminders used” and item #16 “Report multiple ‘denominators’ and provide definitions.” These two items were reported only by 5 of the 8 primary studies. Essential items #1a “Identify mode of delivery in the title” and item #4a “Mention how participants were recruited” were the next least reported items, this information was specified by 6 of the 7 studies.

Regarding the quality of the evidence, four of the nine included articles were considered of moderate quality, three of the primary studies (42, 44, 45) and the systematic review (43). The remaining five studies were weighted as being of low or very low quality according to GRADE (37–41).

### Targeted population

Included studies reported interventions that involved a total of 873 participants with neurodevelopmental disabilities, including ASD.

The total number of involved autistic participants was 164 (96 of those were boys; 38 girls; and in 30 cases the gender of participants was unspecified). Participants were between 5 and 22 years old. Two of these studies specifically included only autistic children with a mean age of 5.5 (in the experimental group) and 5.9 (in the control group) (44) and 8.3 (42); six studies included both autistic children and adolescents (aged 5 to 19) (38–41, 43, 45); and one study specifically focused on autistic high school youths/students (aged 19 to 22) (37).

### Interventions

Four of the studies focused on increasing physical activity and referred to two different interventions (38, 40, 41, 44); while the other studies focused on educating on how to brush teeth (45); educating on safety and social skills on social media (37); improving occupational performance (42); providing information and social support (43); and training social skills (39).

Five studies reported the use of Facebook in the interventions, including Facebook private groups and Facebook secret groups (37, 39–41, 43); three publications used WhatsApp (38, 42, 43); one used YouTube (45); and one study used both WhatsApp and YouTube (44).

Seven articles reported that the duration of the interventions was between 3 and 8 weeks (38–42, 44, 45). The total duration of the intervention package was 315 min in one study (37). The systematic review reported several durations of the interventions, ranging from 6 h to 79 months (43).

Among the primary studies, three articles reported the implementation of two BCW components (37, 39, 42); three articles reported the use of three components (40, 41, 45); and two articles the implementation of seven components (38, 44). The most commonly implemented BCW components were education, which was used by all included primary studies (37–42, 44, 45); followed by training, used in six studies (37–39, 42, 44, 45), and modeling, used in six studies too (38, 40–42, 44, 45).

### Effects of the interventions

Studies weighted as being of moderate quality of evidence have reported significant positive effects in the groups that received the social media interventions: positive influence on social engagement and participation in life situations (43); increased physical activity level (44); increased improvement on occupational performance, specified goals, and behavioral problems (42); and decreased plaque scores coupled with parent reports of intervention success (45).

Four of the five studies weighted as lower quality of evidence reported that the autistic participants and their parents were satisfied with the contents and formats as effects of the social media interventions (38–41). One of these studies also reported no differences in social responsiveness or social skills linked to the social media intervention (39). And one study reported an increase in social media safety skills that was maintained after 21 days (37).

None of the studies included in this review have reported any negative effect linked to social media interventions.

## Discussion

### Summary of findings

Our review found that there is very little evidence of good quality on the use of social media in interventions aimed at autistic individuals. This review includes a total of nine articles: eight primary studies (five non-randomized interventions and three randomized interventions) and one systematic review. The total number of autistic participants that were included in these studies was 164 (5 to 22 years old). The primary studies’ interventions lasted between 3 and 8 weeks, and all of them implemented the education component in their intervention. Six primary studies also implemented training and/or modeling. The studies weighted as being of moderate quality of evidence have reported significant positive effects in the groups that received the social media interventions: increased social engagement and participation in life situations; increased physical activity level; increased improvement in the occupational performance, specified goals, and behavioral problems; and decreased dental plaque scores coupled with parent reports of intervention success. None of the studies have reported any negative effects linked to social media interventions.

### Are social media interventions beneficial for autistic individuals?

There is very little evidence of good quality on the use of social media in interventions aimed at autistic individuals. The number of studies that fulfilled the inclusion criteria and could be included in the review was relatively small, with one review and eight intervention studies. Of the intervention studies, only three were randomized controlled trials. This resulted in an overall quality of evidence level from very low to moderate.

While there is obviously a need for more high-quality studies, all the included studies, with one exception (39), found positive results of the interventions. These findings are encouraging, suggesting that social media-based interventions may in fact be useful for supporting behavioral changes in autistic individuals.

The three included primary studies of the highest methodological quality, the RCTs, all showed important results for health in the intervention groups, i.e., reduced plaque formation (45), an increased improvement in occupational performance, specified goals, and behavioral problems (42), and increased physical activity (44), respectively. While the evidence necessarily is limited by the low number of RCTs, these findings suggest that social media interventions can be used to deliver interventions that can improve the health of autistic individuals– a group that might not be able to benefit from the same degree from health interventions directed to the general public.

It is important to have in mind that autistic individuals can have very different types and levels of challenges. This group of people includes individuals that can be at a high intellectual level but struggle in other areas, as well as individuals with severe intellectual disabilities (46). As a consequence, a specific social media-based intervention for autistic individuals should perhaps be targeted to and be suited only for a sub-group of people with ASD and/or their caregivers. However, studies included in this review do not allude to the severity of ASD symptomology or challenges with regard to their usage of social media as a behavioral intervention. Further research could explore the effect of social media interventions in autistic individuals with different levels of challenges.

### Knowledge gaps and future directions

Despite the widespread use of social media among individuals, including autistic individuals (25, 26, 29, 30), research using social media in interventions for autism is currently very limited. Most of the included studies have been addressed to caregivers or educators of autistic children or young individuals. Few interventions are directly addressed to autistic adults. Since ASD is a lifelong developmental condition, future research could explore the potential benefits of addressing social media interventions to autistic adults and to involve recipients of such interventions in both the identification of areas suitable for interventions, and in study design (29).

Nearly all the included studies have used restricted-access social media, such as Facebook secret or private groups, or WhatsApp. These types of media provide better control of the environment for research purposes, but also help to protect individuals’ privacy. Further research could also explore the potential of using additional social media channels in which study participants could feel safe and comfortable (30).

Interventions for autistic individuals are complex, and identifying what are the effective or ineffective components is challenging. A poor description and reporting of intervention studies makes evaluation difficult. As exemplified, in this review we found that none of the included primary studies reported on one of the essential 17 items required by CONSORT-EHEALTH (item #12 “Imputation techniques to deal with attrition or missing values”) (35). Reporting on the use or not use of prompts/reminders (item #5), or the description of multiple denominators (item #16) was only stated in five of the publications. Future research using social media or other digital technology in their interventions are encouraged to adhere to CONSORT-EHEALTH standards (35) and to report all recommended items in their publications. An adequate description of the methods and interventions used in research will help to identify the effective components, and will also allow other researchers to replicate the study. Since most social media are of free use, the identification of successful interventions delivered through these channels could potentially help to extend these successful interventions to other individuals worldwide, including the developing world. Social media interventions can also be used to prevent misinformation about autism, and to spread important health information to both autistic persons and the general public (27, 28). This can prevent myths and stereotypical views about autism to take hold.

### Study limitations

Our review has several limitations. Although our search covered several databases and keywords and probably most of the available research on this field has been identified; we might have missed relevant publications. We did not explore the gray literature, and a total of 10 studies specifically focusing on social media interventions for autism were excluded for not providing results at the time when this review was done. However, this suggests that additional evidence on social media interventions for ASD may be published in the near future.

We were not able to conduct a meta-analysis because of the small number of studies and the diverse nature of these studies. Only nine studies were included in this review. The evidence is limited, and in addition, the quality of the included studies is low, which is a strong limitation. Caution should be noted with regard to study findings due to the limited number of retrieved studies and low quality.

## Conclusion

Our review found that there is very little evidence of good quality on the use of social media in interventions aimed at autistic individuals. While there is obviously a need for more high-quality studies, all the included studies, with one exception, found positive results of the interventions. These findings are encouraging, suggesting that social media-based interventions may in fact be useful for supporting behavioral changes in autistic individuals.

## Data availability statement

The original contributions presented in this study are included in the article/Supplementary material, further inquiries can be directed to the corresponding author.

## Author contributions

EG: conception and design of the review, database search, and first draft. EG, IS, and AN-H: title, abstract, and full text screening. EG and AN-H: data extraction and verification. EG and RW: risk of bias assessment and coding and verification. EG, IS, and RW: drafting additional versions of the review. All authors approved the final version of the manuscript.
