# Supplementary material for: Social media interventions for autistic individuals: Systematic review
Source: Front Psychiatry. 2023 Mar 1;14:1089452. doi: 10.3389/fpsyt.2023.1089452 (PMC10014977; doi:10.3389/fpsyt.2023.1089452)
Supplement: Supplementary file 1 [file Data_Sheet_1.pdf]

Appendix 1. Full search strategy

First search: 4th July 2022

Database	**Search engine**	Entries
PubMed	**S1** (((((((((((((((((((((Social media[Title/Abstract]) OR (Social networking[Title/Abstract])) OR (Facebook[Title/Abstract])) OR (YouTube[Title/Abstract])) OR (WhatsApp[Title/Abstract])) OR (Messenger[Title/Abstract])) OR (Instagram[Title/Abstract])) OR (WeChat[Title/Abstract])) OR (Kuaishou[Title/Abstract])) OR (TikTok[Title/Abstract])) OR (Telegram[Title/Abstract])) OR (Qzone[Title/Abstract])) OR (QQ[Title/Abstract])) OR (Weibo[Title/Abstract])) OR (Douyin[Title/Abstract])) OR (Snapchat[Title/Abstract])) OR (Twitter[Title/Abstract])) OR (Pinterest[Title/Abstract])) OR (Reddit[Title/Abstract])) OR (LinkedIn[Title/Abstract])) OR (Quora[Title/Abstract])) OR (Skype[Title/Abstract])<br><br>**S2** ((((((((((Autism Spectrum Disorder[Title/Abstract]) OR (Autistic Disorder[Title/Abstract])) OR (Autism[Title/Abstract])) OR (Autistic[Title/Abstract])) OR (ASD[Title/Abstract])) OR (Asperger Syndrome[Title/Abstract])) OR (Asperger[Title/Abstract])) OR (Pervasive developmental disorder[Title/Abstract])) OR (Pervasive development disorder[Title/Abstract])) OR (PDD[Title/Abstract])) OR (PDD-NOS[Title/Abstract])<br><br>**S1 AND S2**	19
EMBASE	**S1** (Social media or Social networking or Facebook or YouTube or WhatsApp or Messenger or Instagram or WeChat or Kuaishou or TikTok or Telegram or Qzone or QQ or Weibo or Douyin or Snapchat or Twitter or Pinterest or Reddit or LinkedIn or Quora or Skype).ti.<br><br>**S2** (Autism Spectrum Disorder or Autistic Disorder or Autism or Autistic or ASD or Asperger Syndrome or Asperger or Pervasive developmental disorder or Pervasive development disorder or PDD or PDD-NOS).ti.<br><br>**S1 AND S2**	40
EMBASE	**S1** (Social media or Social networking or Facebook or YouTube or WhatsApp or Messenger or Instagram or WeChat or Kuaishou or TikTok or Telegram or Qzone or QQ or Weibo or Douyin or Snapchat or Twitter or Pinterest or Reddit or LinkedIn or Quora or Skype).ab.<br><br>**S2** (Autism Spectrum Disorder or Autistic Disorder or Autism or Autistic or ASD or Asperger Syndrome or Asperger or Pervasive developmental disorder or Pervasive development disorder or PDD or PDD-NOS).ab.<br><br>**S1 AND S2**	312
Cochrane Library	**S1** Social media or Social networking or Facebook or YouTube or WhatsApp or Messenger or Instagram or WeChat or Kuaishou or TikTok or Telegram or Qzone or QQ or Weibo or Douyin or Snapchat or Twitter or Pinterest or Reddit or LinkedIn or Quora or Skype:ti,ab,kw	200

	**S2** Autism Spectrum Disorder or Autistic Disorder or Autism or Autistic or ASD or Asperger Syndrome or Asperger or Pervasive developmental disorder or Pervasive development disorder or PDD or PDD-NOS:ti,ab,kw<br><br>**S1 AND S2**	
PyscInfo	**S1** (Social media or Social networking or Facebook or YouTube or WhatsApp or Messenger or Instagram or WeChat or Kuaishou or TikTok or Telegram or Qzone or QQ or Weibo or Douyin or Snapchat or Twitter or Pinterest or Reddit or LinkedIn or Quora or Skype).ti.<br><br>**S2** (Autism Spectrum Disorder or Autistic Disorder or Autism or Autistic or ASD or Asperger Syndrome or Asperger or Pervasive developmental disorder or Pervasive development disorder or PDD or PDD-NOS).ti.<br><br>**S1 AND S2**	31
PyscInfo	**S1** (Social media or Social networking or Facebook or YouTube or WhatsApp or Messenger or Instagram or WeChat or Kuaishou or TikTok or Telegram or Qzone or QQ or Weibo or Douyin or Snapchat or Twitter or Pinterest or Reddit or LinkedIn or Quora or Skype).ab.<br><br>**S2** (Autism Spectrum Disorder or Autistic Disorder or Autism or Autistic or ASD or Asperger Syndrome or Asperger or Pervasive developmental disorder or Pervasive development disorder or PDD or PDD-NOS).ab.<br><br>**S1 AND S2**	173
ERIC	**S1** TI Social media OR TI Social networking OR TI Facebook OR TI YouTube OR TI WhatsApp OR TI Messenger OR TI Instagram OR TI WeChat OR TI Kuaishou OR TI TikTok OR TI Telegram OR TI Qzone TI Weibo OR TI Douyin OR TI Snapchat OR TI Twitter OR TI Pinterest OR TI Reddit OR TI LinkedIn OR TI Quora OR TI Skype<br><br>**S2** TI Autism Spectrum Disorder OR TI Autistic Disorder OR TI Autism OR TI Autistic OR TI ASD OR TI Asperger Syndrome OR TI Asperger OR TI Pervasive developmental disorder OR TI Pervasive development disorder OR TI PDD OR TI PDD-NOS<br><br>(TI Autism Spectrum Disorder OR TI Autistic Disorder OR TI Autism OR TI Autistic OR TI ASD OR TI Asperger Syndrome OR TI Asperger OR TI Pervasive developmental disorder OR TI Pervasive development disorder OR TI PDD OR TI PDD-NOS) AND (S1 OR S2)	10
ERIC	**S1** AB Social media OR AB Social networking OR AB Facebook OR AB YouTube OR AB WhatsApp OR AB Messenger OR AB Instagram OR AB WeChat OR AB Kuaishou OR AB TikTok OR AB Telegram OR AB Qzone<br>AB Weibo OR AB Douyin OR AB Snapchat OR AB Twitter OR AB Pinterest OR AB Reddit OR AB LinkedIn OR AB Quora OR AB Skype<br><br>**S2** AB Autism Spectrum Disorder OR AB Autistic Disorder OR AB Autism OR AB Autistic OR AB ASD OR AB Asperger Syndrome OR AB Asperger OR AB Pervasive developmental disorder OR AB Pervasive development disorder OR AB PDD OR AB PDD-NOS	41

	(AB Autism Spectrum Disorder OR AB Autistic Disorder OR AB Autism OR AB Autistic OR AB ASD OR AB Asperger Syndrome OR AB Asperger OR AB Pervasive developmental disorder OR AB Pervasive development disorder OR AB PDD OR AB PDD-NOS) AND (S1 OR S2)	
Education Source	**S1** TI Social media OR TI Social networking OR TI Facebook OR TI YouTube OR TI WhatsApp OR TI Messenger OR TI Instagram OR TI WeChat OR TI Kuaishou OR TI TikTok OR TI Telegram OR TI Qzone TI Weibo OR TI Douyin OR TI Snapchat OR TI Twitter OR TI Pinterest OR TI Reddit OR TI LinkedIn OR TI Quora OR TI Skype<br><br>**S2** TI Autism Spectrum Disorder OR TI Autistic Disorder OR TI Autism OR TI Autistic OR TI ASD OR TI Asperger Syndrome OR TI Asperger OR TI Pervasive developmental disorder OR TI Pervasive development disorder OR TI PDD OR TI PDD-NOS<br><br>(TI Autism Spectrum Disorder OR TI Autistic Disorder OR TI Autism OR TI Autistic OR TI ASD OR TI Asperger Syndrome OR TI Asperger OR TI Pervasive developmental disorder OR TI Pervasive development disorder OR TI PDD OR TI PDD-NOS) AND (S1 OR S2)	20
Education Source	**S1** AB Social media OR AB Social networking OR AB Facebook OR AB YouTube OR AB WhatsApp OR AB Messenger OR AB Instagram OR AB WeChat OR AB Kuaishou OR AB TikTok OR AB Telegram OR AB Qzone<br>AB Weibo OR AB Douyin OR AB Snapchat OR AB Twitter OR AB Pinterest OR AB Reddit OR AB LinkedIn OR AB Quora OR AB Skype<br><br>**S2** AB Autism Spectrum Disorder OR AB Autistic Disorder OR AB Autism OR AB Autistic OR AB ASD OR AB Asperger Syndrome OR AB Asperger OR AB Pervasive developmental disorder OR AB Pervasive development disorder OR AB PDD OR AB PDD-NOS<br><br>(AB Autism Spectrum Disorder OR AB Autistic Disorder OR AB Autism OR AB Autistic OR AB ASD OR AB Asperger Syndrome OR AB Asperger OR AB Pervasive developmental disorder OR AB Pervasive development disorder OR AB PDD OR AB PDD-NOS) AND (S1 OR S2)	72
Web of Science	**S1** ((((((((((((((((((((TI=(Social media)) OR TI=(Social networking)) OR TI=(Facebook)) OR TI=(YouTube)) OR TI=(WhatsApp)) OR TI=(Messenger)) OR TI=(Instagram)) OR TI=(WeChat)) OR TI=(Kuaishou)) OR TI=(TikTok)) OR TI=(Telegram)) OR TI=(Qzone)) OR TI=(QQ)) OR TI=(Weibo)) OR TI=(Douyin)) OR TI=(Snapchat)) OR TI=(Twitter)) OR TI=(Pinterest)) OR TI=(Reddit)) OR TI=(LinkedIn)) OR TI=(Quora)) OR TI=(Skype)<br><br>**S2** (((((((((TI=(Autism Spectrum Disorder)) OR TI=(Autistic Disorder)) OR TI=(Autism)(Prandini et al.)) OR TI=(Autistic)) OR TI=(ASD)) OR TI=(Asperger Syndrome)) OR TI=(Asperger)) OR TI=(Pervasive Developmental Disorder)) OR TI=(Pervasive Development Disorder)) OR TI=(PDD)) OR TI=(PDD-NOS)<br><br>**S1 AND S2**	135
Web of Science	**S1** ((((((((((((((((((((AB=(Social media)) OR AB=(Social networking)) OR AB=(Facebook)) OR AB=(YouTube)) OR AB=(WhatsApp)) OR AB=(Messenger)) OR AB=(Instagram)) OR AB=(WeChat)) OR AB=(Kuaishou)) OR AB=(TikTok)) OR AB=(Telegram)) OR	1567

	AB=(Qzone)) OR AB=(QQ)) OR AB=(Weibo)) OR AB=(Douyin)) OR AB=(Snapchat)) OR AB=(Twitter)) OR AB=(Pinterest)) OR AB=(Reddit)) OR AB=(LinkedIn)) OR AB=(Quora)) OR AB=(Skype)<br><br>**S2** ((((((((((AB=(Autism Spectrum Disorder)) OR AB=(Autistic Disoder)) OR AB=(Autism)(Prandini et al.)) OR AB=(Autistic)) OR AB=(ASD)) OR AB=(Asperger Syndrome)) OR AB=(Asperger)) OR AB=(Pervasive Developmental Disorder)) OR AB=(Pervasive Development Disorder)) OR AB=(PDD)) OR AB=(PDD-NOS)<br><br>**S1 AND S2**	
IEEE Xplore	(((((No Keywords Specified))) AND ((Document Title:Social media) OR (Document Title:Social networking) OR (Document Title:Facebook) OR (Document Title:YouTube) OR (Document Title:WhatsApp) OR (Document Title:Messenger) OR (Document Title:Instagram) OR (Document Title:WeChat) OR (Document Title:Kuaishou) OR (Document Title:TikTok) OR (Document Title:Telegram))) OR ((Document Title:Qzone) OR (Document Title:QQ) OR (Document Title:Weibo) OR (Document Title:Douyin) OR (Document Title:Snapchat) OR (Document Title:Twitter) OR (Document Title:Pinterest) OR (Document Title:Reddit) OR (Document Title:LinkedIn) OR (Document Title:Quora) OR (Document Title:Skype))) AND ((Document Title:Autism Spectrum Disorder) OR (Document Title:Autistic Disorder) OR (Document Title:Autism) OR (Document Title:Autistic) OR (Document Title:ASD) OR (Document Title:Asperger Syndrome) OR (Document Title:Asperger) OR (Document Title:Pervasive developmental disorder) OR (Document Title:Pervasive development disorder) OR (Document Title:PDD) OR (Document Title:PDD-NOS))	7
IEEE Xplore	(((((No Keywords Specified))) AND ((Abstract:Social media) OR (Abstract:Social networking) OR (Abstract:Facebook) OR (Abstract:YouTube) OR (Abstract:WhatsApp) OR (Abstract:Messenger) OR (Abstract:Instagram) OR (Abstract:WeChat) OR (Abstract:Kuaishou) OR (Abstract:TikTok) OR (Abstract:Telegram))) OR ((Abstract:Qzone) OR (Abstract:QQ) OR (Abstract:Weibo) OR (Abstract:Douyin) OR (Abstract:Snapchat) OR (Abstract:Twitter) OR (Abstract:Pinterest) OR (Abstract:Reddit) OR (Abstract:LinkedIn) OR (Abstract:Quora) OR (Abstract:Skype))) AND ((Abstract:Autism Spectrum Disorder) OR (Abstract:Autistic Disorder) OR (Abstract:Autism) OR (Abstract:Autistic) OR (Abstract:ASD) OR (Abstract:Asperger Syndrome) OR (Abstract:Asperger) OR (Abstract:Pervasive developmental disorder) OR (Abstract:Pervasive development disorder) OR (Abstract:PDD) OR (Abstract:PDD-NOS))	100
	Total (including duplicates)	2727

Second search: 4th October 2022

Database (publication time limit)	Search engine	Entries
PubMed (2022/7/5 - 2022/10/4)	**S1** (((((((((((((((((((((Social media[Title/Abstract]) OR (Social networking[Title/Abstract])) OR (Facebook[Title/Abstract])) OR (YouTube[Title/Abstract])) OR (WhatsApp[Title/Abstract])) OR (Messenger[Title/Abstract])) OR (Instagram[Title/Abstract])) OR (WeChat[Title/Abstract])) OR (Kuaishou[Title/Abstract])) OR (TikTok[Title/Abstract])) OR (Telegram[Title/Abstract])) OR (Qzone[Title/Abstract])) OR (QQ[Title/Abstract])) OR (Weibo[Title/Abstract])) OR (Douyin[Title/Abstract])) OR (Snapchat[Title/Abstract])) OR (Twitter[Title/Abstract])) OR (Pinterest[Title/Abstract])) OR (Reddit[Title/Abstract])) OR (LinkedIn[Title/Abstract])) OR (Quora[Title/Abstract])) OR (Skype[Title/Abstract])<br><br>**S2** ((((((((((Autism Spectrum Disorder[Title/Abstract]) OR (Autistic Disorder[Title/Abstract])) OR (Autism[Title/Abstract])) OR (Autistic[Title/Abstract])) OR (ASD[Title/Abstract])) OR (Asperger Syndrome[Title/Abstract])) OR (Asperger[Title/Abstract])) OR (Pervasive developmental disorder[Title/Abstract])) OR (Pervasive development disorder[Title/Abstract])) OR (PDD[Title/Abstract])) OR (PDD-NOS[Title/Abstract])<br><br>**S1 AND S2**	11
EMBASE (2022-Current)	**S1** ((Social media or Social networking or Facebook or YouTube or WhatsApp or Messenger or Instagram or WeChat or Kuaishou or TikTok or Telegram or Qzone or QQ or Weibo or Douyin or Snapchat or Twitter or Pinterest or Reddit or LinkedIn or Quora or Skype)<br>AND<br>(Autism Spectrum Disorder or Autistic Disorder or Autism or Autistic or ASD or Asperger Syndrome or Asperger or Pervasive developmental disorder or Pervasive development disorder or PDD or PDD-NOS)).ti.<br><br>**S2**<br>((Social media or Social networking or Facebook or YouTube or WhatsApp or Messenger or Instagram or WeChat or Kuaishou or TikTok or Telegram or Qzone or QQ or Weibo or Douyin or Snapchat or Twitter or Pinterest or Reddit or LinkedIn or Quora or Skype)<br>AND<br>(Autism Spectrum Disorder or Autistic Disorder or Autism or Autistic or ASD or Asperger Syndrome or Asperger or Pervasive developmental disorder or Pervasive development disorder or PDD or PDD-NOS)).ab.<br><br>**S1 OR S2**	49
Cochrane Library (2022-2022)	**S1** Social media or Social networking or Facebook or YouTube or WhatsApp or Messenger or Instagram or WeChat or Kuaishou or TikTok or Telegram or Qzone or QQ or Weibo or Douyin or Snapchat or Twitter or Pinterest or Reddit or LinkedIn or Quora or Skype:ti,ab,kw	14

	**S2** Autism Spectrum Disorder or Autistic Disorder or Autism or Autistic or ASD or Asperger Syndrome or Asperger or Pervasive developmental disorder or Pervasive development disorder or PDD or PDD-NOS:ti,ab,kw<br><br>**S1 AND S2**	
PyscInfo (2022-Current)	**S1** (Social media or Social networking or Facebook or YouTube or WhatsApp or Messenger or Instagram or WeChat or Kuaishou or TikTok or Telegram or Qzone or QQ or Weibo or Douyin or Snapchat or Twitter or Pinterest or Reddit or LinkedIn or Quora or Skype).ti.<br>AND<br>(Autism Spectrum Disorder or Autistic Disorder or Autism or Autistic or ASD or Asperger Syndrome or Asperger or Pervasive developmental disorder or Pervasive development disorder or PDD or PDD-NOS).ti.<br><br>**S2** (Social media or Social networking or Facebook or YouTube or WhatsApp or Messenger or Instagram or WeChat or Kuaishou or TikTok or Telegram or Qzone or QQ or Weibo or Douyin or Snapchat or Twitter or Pinterest or Reddit or LinkedIn or Quora or Skype).ab.<br>AND<br>(Autism Spectrum Disorder or Autistic Disorder or Autism or Autistic or ASD or Asperger Syndrome or Asperger or Pervasive developmental disorder or Pervasive development disorder or PDD or PDD-NOS).ab.<br><br>**S1 OR S2**	12
ERIC (July 2022-October 2022)	**S1** (TI Social media OR TI Social networking OR TI Facebook OR TI YouTube OR TI WhatsApp OR TI Messenger OR TI Instagram OR TI WeChat OR TI Kuaishou OR TI TikTok OR TI Telegram OR TI Qzone TI Weibo OR TI Douyin OR TI Snapchat OR TI Twitter OR TI Pinterest OR TI Reddit OR TI LinkedIn OR TI Quora OR TI Skype)<br>AND<br>(TI Autism Spectrum Disorder OR TI Autistic Disorder OR TI Autism OR TI Autistic OR TI ASD OR TI Asperger Syndrome OR TI Asperger OR TI Pervasive developmental disorder OR TI Pervasive development disorder OR TI PDD OR TI PDD-NOS)<br><br>**S2** (AB Social media OR AB Social networking OR AB Facebook OR AB YouTube OR AB WhatsApp OR AB Messenger OR AB Instagram OR AB WeChat OR AB Kuaishou OR AB TikTok OR AB Telegram OR AB Qzone<br>AB Weibo OR AB Douyin OR AB Snapchat OR AB Twitter OR AB Pinterest OR AB Reddit OR AB LinkedIn OR AB Quora OR AB Skype)<br>AND<br>(AB Autism Spectrum Disorder OR AB Autistic Disorder OR AB Autism OR AB Autistic OR AB ASD OR AB Asperger Syndrome OR AB Asperger OR AB Pervasive developmental disorder OR AB Pervasive development disorder OR AB PDD OR AB PDD-NOS)<br><br>**S1 OR S2**	0
Education Source (July 2022-	**S1** (TI Social media OR TI Social networking OR TI Facebook OR TI YouTube OR TI WhatsApp OR TI Messenger OR TI Instagram OR TI WeChat OR TI Kuaishou OR TI TikTok OR TI Telegram OR TI Qzone	1

October 2022)	TI Weibo OR TI Douyin OR TI Snapchat OR TI Twitter OR TI Pinterest OR TI Reddit OR TI LinkedIn OR TI Quora OR TI Skype)<br>AND<br>(TI Autism Spectrum Disorder OR TI Autistic Disorder OR TI Autism OR TI Autistic OR TI ASD OR TI Asperger Syndrome OR TI Asperger OR TI Pervasive developmental disorder OR TI Pervasive development disorder OR TI PDD OR TI PDD-NOS)<br><br>**S2** (AB Social media OR AB Social networking OR AB Facebook OR AB YouTube OR AB WhatsApp OR AB Messenger OR AB Instagram OR AB WeChat OR AB Kuaishou OR AB TikTok OR AB Telegram OR AB Qzone<br>AB Weibo OR AB Douyin OR AB Snapchat OR AB Twitter OR AB Pinterest OR AB Reddit OR AB LinkedIn OR AB Quora OR AB Skype)<br>AND<br>(AB Autism Spectrum Disorder OR AB Autistic Disorder OR AB Autism OR AB Autistic OR AB ASD OR AB Asperger Syndrome OR AB Asperger OR AB Pervasive developmental disorder OR AB Pervasive development disorder OR AB PDD OR AB PDD-NOS)<br><br>**S1 OR S2**	
Web of Science (2022)	**S1** ((((((((((((((((((((TI=(Social media)) OR TI=(Social networking)) OR TI=(Facebook)) OR TI=(YouTube)) OR TI=(WhatsApp)) OR TI=(Messenger)) OR TI=(Instagram)) OR TI=(WeChat)) OR TI=(Kuaishou)) OR TI=(TikTok)) OR TI=(Telegram)) OR TI=(Qzone)) OR TI=(QQ)) OR TI=(Weibo)) OR TI=(Douyin)) OR TI=(Snapchat)) OR TI=(Twitter)) OR TI=(Pinterest)) OR TI=(Reddit)) OR TI=(LinkedIn)) OR TI=(Quora)) OR TI=(Skype)<br>AND<br>(((((((((TI=(Autism Spectrum Disorder)) OR TI=(Autistic Disoder)) OR TI=(Prandini et al.)) OR TI=(Autistic)) OR TI=(ASD)) OR TI=(Asperger Syndrome)) OR TI=(Asperger)) OR TI=(Pervasive Developmental Disorder)) OR TI=(Pervasive Development Disorder)) OR TI=(PDD)) OR TI=(PDD-NOS)<br><br>**S2** ((((((((((((((((((((AB=(Social media)) OR AB=(Social networking)) OR AB=(Facebook)) OR AB=(YouTube)) OR AB=(WhatsApp)) OR AB=(Messenger)) OR AB=(Instagram)) OR AB=(WeChat)) OR AB=(Kuaishou)) OR AB=(TikTok)) OR AB=(Telegram)) OR AB=(Qzone)) OR AB=(QQ)) OR AB=(Weibo)) OR AB=(Douyin)) OR AB=(Snapchat)) OR AB=(Twitter)) OR AB=(Pinterest)) OR AB=(Reddit)) OR AB=(LinkedIn)) OR AB=(Quora)) OR AB=(Skype)<br>AND<br>(((((((((AB=(Autism Spectrum Disorder)) OR AB=(Autistic Disoder)) OR AB=(Autism)) OR AB=(Autistic)) OR AB=(ASD)) OR AB=(Asperger Syndrome)) OR AB=(Asperger)) OR AB=(Pervasive Developmental Disorder)) OR AB=(Pervasive Development Disorder)) OR AB=(PDD)) OR AB=(PDD-NOS)<br><br>**S1 OR S2**	175
IEEE Xplore	(((((No Keywords Specified))) AND ((Document Title:Social media) OR (Document Title:Social networking) OR (Document Title:Facebook) OR (Document Title:YouTube) OR (Document Title:WhatsApp) OR (Document Title:Messenger) OR (Document Title:Instagram) OR (Document Title:WeChat) OR (Document	0

	Title:Kuaishou) OR (Document Title:TikTok) OR (Document Title:Telegram))) OR ((Document Title:Qzone) OR (Document Title:QQ) OR (Document Title:Weibo) OR (Document Title:Douyin) OR (Document Title:Snapchat) OR (Document Title:Twitter) OR (Document Title:Pinterest) OR (Document Title:Reddit) OR (Document Title:LinkedIn) OR (Document Title:Quora) OR (Document Title:Skype))) AND ((Document Title:Autism Spectrum Disorder) OR (Document Title:Autistic Disorder) OR (Document Title:Autism) OR (Document Title:Autistic) OR (Document Title:ASD) OR (Document Title:Asperger Syndrome) OR (Document Title:Asperger) OR (Document Title:Pervasive developmental disorder) OR (Document Title:Pervasive development disorder) OR (Document Title:PDD) OR (Document Title:PDD-NOS))	
IEEE Xplore	(((((No Keywords Specified))) AND ((Abstract:Social media) OR (Abstract:Social networking) OR (Abstract:Facebook) OR (Abstract:YouTube) OR (Abstract:WhatsApp) OR (Abstract:Messenger) OR (Abstract:Instagram) OR (Abstract:WeChat) OR (Abstract:Kuaishou) OR (Abstract:TikTok) OR (Abstract:Telegram))) OR ((Abstract:Qzone) OR (Abstract:QQ) OR (Abstract:Weibo) OR (Abstract:Douyin) OR (Abstract:Snapchat) OR (Abstract:Twitter) OR (Abstract:Pinterest) OR (Abstract:Reddit) OR (Abstract:LinkedIn) OR (Abstract:Quora) OR (Abstract:Skype))) AND ((Abstract:Autism Spectrum Disorder) OR (Abstract:Autistic Disorder) OR (Abstract:Autism) OR (Abstract:Autistic) OR (Abstract:ASD) OR (Abstract:Asperger Syndrome) OR (Abstract:Asperger) OR (Abstract:Pervasive developmental disorder) OR (Abstract:Pervasive development disorder) OR (Abstract:PDD) OR (Abstract:PDD-NOS))	0
	Total (including duplicates)	262

Appendix 2. List of relevant studies that were excluded in the full-text review and reasons for exclusion

Article	Reasons for rejection			
	Not social media use	Not dealing with autism	Not an intervention for ASD	Not reporting results
Bilke-Hentsch, O. and Hartmann, M. (2015), 'Digital technologies and cyberculture in in-patient psychiatric treatment of adolescents: A technology based therapeutic and pedagogic intervention (Somosa Medialabr)', *European Child and Adolescent Psychiatry,* 24 (1 SUPPL. 1), S245			X	
Brown, S. and Doherty, M. (2021), 'Raising awareness of support available to autistic anaesthetists: Autistic Doctors International', *Anaesthesia,* 76 (SUPPL 2), 25.				X
Caton, S. and Landman, R. (2019), 'Increasing awareness of online radicalisation for young people with intellectual disabilities: Evaluating the get smart (social media awareness and resilience training) project', *Journal of Intellectual Disability Research,* 63 (7), 657	X			
Ceranoglu, T. A. (IRCT20160808029255N2), 'Autism spectrum disorders and electronic media use: Empathy with avatars', *Journal of the American Academy of Child and Adolescent Psychiatry,* 55 (10 Supplement 1), S70			X	
Ctri (2021), 'A study to compare therapy through mobile phone internet vs. therapy in-person for children with problems of development', *https://trialsearch.who.int/Trial2.aspx?TrialID=CTRI/2021/12/038600*				X
Curtiss, S.L.; Ebata A.T. (IRCT20160808029255N2), 'Building Capacity to Deliver Sex Education to Individuals with Autism'. *Sexuality Disability,* 34:27–47			X	
Galbraith, Carolyn and Lancaster, Julie (2020), 'Children with Autism in Wild Nature: Exploring Australian Parent Perceptions Using Photovoice', *Journal of Outdoor and Environmental Education,* 23 (3), 293-307			X	
García-Villamisar, D. A. and Dattilo, J. (IRCT20220107053651N1), 'Effects of a leisure programme on quality of life and stress of individuals with ASD', *Journal of intellectual disability research,* 54 (7), 611-19	X			
Gwynette, M. F. (IRCT20160808029255N2), 'Redefining social skills training in autism spectrum disorders: facebook and other applications', *Journal of the american academy of child and adolescent psychiatry,* 55 (10), S71				X
Irct20200922048797N (2020a), 'The effect of educational intervention based on Social Cognitive Theory on Quality of Life among parents of Autistic patients', *https://trialsearch.who.int/Trial2.aspx?TrialID=IRCT20200922048797N1*				X
Irct20200208046413N (2020b), 'Evaluation of an oral hygiene educational intervention to improve the oral health of elementary students with autism', *https://trialsearch.who.int/Trial2.aspx?TrialID=IRCT20200208046413N1*				X

Irwandy, D., et al. (2021), 'Creative Learning Media for High-Functioning Autistic Student Using Video Sharing Platform during the Pandemic', *2021 International Conference on Information Management and Technology (ICIMTech)* (1), 12-17.	X			
Marchand, Geneviève and Healy, Sean (2019), 'Implementation of Project CHASE (Children with Autism Supported to Exercise): A Facebook-Delivered, Parent-Mediated Physical Activity Intervention', *Palaestra,* 33 (3), 32-39				X
Nct (2013), 'Using Video Modeled Social Stories to Improve Oral Hygiene in Children With Autism Spectrum Disorder', *https://clinicaltrials.gov/show/NCT02003820*				X
--- (2015a), 'ASD Parent Trainer: Online Coaching for Parents of Children With Autism', *https://clinicaltrials.gov/show/NCT02469870*	X			
Nct (2015b), 'Efficacy of Training Programme to Reduce Stress', *https://clinicaltrials.gov/show/NCT02384486*				X
Nct (IRCT20160808029255N2), 'Facebook for Social Skills Training in Autism: Project Rex Connect', *https://clinicaltrials.gov/show/NCT02897687*				X
Nct (2021), 'Fit Families Program for Families of Children With Autism Spectrum Disorder', *https://clinicaltrials.gov/show/NCT05159102*				X
Probst, Donnell (2017), 'Social Media Literacy as an IEP Intervention for Social and Emotional Learning', *Journal of Media Literacy Education,* 9 (2), 45-57			X	
Rascon, N. A. (2022), 'A Communication Complex Approach to Autism Awareness Training Within First Response Systems in Indiana', *FRONTIERS IN COMMUNICATION,* 7	X			
Sidhu, S. S., Gwynette, M. F., and Weele, J. M. V. V. (IRCT20160808029255N2), 'Will you friend me? understanding the complex interplay between social media, online gaming, and technology in autism spectrum disorder', *Journal of the American Academy of Child and Adolescent Psychiatry,* 55 (10 Supplement 1), S70			X	
